# A General System of Toxicology, or a Treatise on Poisons Drawn from the Mineral, Vegetable, and Animal Kingdoms, &c.

**Published:** 1816-06

**Authors:** 


*Traité des Poisons tirés des Regnes Minéral, Végétal, et Animal, ou Toxicologie Générale, &c.* Par M. P. Orfila, D. M. &c.

---

*A General System of Toxicology, or a Treatise on Poisons drawn from the Mineral, Vegetable, and Animal Kingdoms, &c.* By M. P. Orfila, M. D. &c. *Translated from the French.* Vol. I. Parts I and and II.

( Continued from page 435. )

We have dwelt so long upon the two first and most interesting articles of this volume, that, in order to preserve our readers from drowsiness or absolute despair, it is proper to announce to them our design of passing more rapidly over the remaining portion.

*Article* 3. *Species* 3. *Antimonial Poisons.*—Out of the seven varieties, here specified, we shall select, as the object of our attention, *Emetic tartar*. It is one of the most powerful and frequently employed preparations of antimony. For obvious reasons, a brief notice of it will suffice.

*Emetic Tartar** is of a tart and metallic taste. Heated to redness in an earthen crucible, it blackens, is decomposed, and yields metallic antimony, and potash slightly carbonated. The same phænomenon will occur, but more quickly, if it be thrown in powder upon live coals. Soluble in somewhat more than fourteen parts of distilled water at the temperature of from 10° to 12° R.† and in less than two parts of boiling water, if quite pure. This solution reddens litmus paper and tincture; furnishes with sulphuretted hydrogen gas and the hydro-sulphurets in small quantity, an orange-yellow precipitate, in excess, a deep red-brown; white with concentrated sulphuric acid, alum, lime-water, barytes-water, and carbonate of soda; reddish-yellow with vegetable juices and decoctions; dirty white bordering upon yellow, with tincture of nut-galls. The latter forms the most delicate test of emetic tartar. But none of these tests are to be depended on if the solution be mixed with *red wine* or *tea*. *Albumen* produces no disturbance in it; and the hydro-sulphurets and nut-gall operate as on the simple solution. With *gelatine*, it is abundantly precipitated by nut-gall, and affected by the other tests as when uncombined. It does not coagulate milk; hydro-sulphuret of ammonia precipitates the mixture of a clear red. Alkaline sulphates, neutral earths, *broth*, and *human bile* do not disturb the solution.—*The action of emetic tartar upon the animal œconomy* is greatly modified by the manner in which the stomach is affected. Persons have recovered after ingestion of the largest doses where vomiting ensued: while others, who experienced not this salutary operation, and animals, in which it was artificially prevented, have been destroyed by the operation of the poison. Ma-

---

* *Tartrate de potasse antimonié,—tartre stibié* of French chemists; *tartrite of antimony*, and *tartarized antimony*, of the British Ph. Thenard's analysis gives tartrate of potash 34,—tartrate of antimony 54,—water 8, loss 4, in the hundred parts. Rev.

† 54—59 Fahrenh. Rev.

gendie, from the light of experiment, infers that it affects principally the pulmonary texture and intestinal mucous membrane; and that its deleterious consequences are referrible rather to absorption than any direct operation on the stomach. The *symptoms* characteristic of this poison, deduced from four well-selected cases by Orfila, are, austere metallic taste; nausea, vomiting, hiccough, cardialgia, burning heat in the epigastrium, pains of the stomach, colic, distension by flatus, copious stools; syncope; small, concentrated, frequent pulse; skin cold or intensely hot; dyspnœa; vertigo, insensibility, convulsive motions, spasms of the legs, exhaustion, death. Deglutition is also sometimes difficult or suspended. *The organic lesions* induced are, inflammation, and sometimes gangrene, of the lungs and intestinal mucous membrane, particularly that of the stomach.—With respect to *the application of preceding facts in different cases of poisoning by emetic tartar*, it will suffice to observe that all the suspected liquids should be examined by the tests before indicated, particularly tincture of litmus, the hydro-sulphurets, nut-gall, sulphuric acid and lime-water; and that attempts should be made to obtain metallic antimony, by calcination, from the various precipitates thus formed, or other solid matters in which the existence of the poison is suspected. It should be remembered that emetic tartar is the only preparation of antimony containing a vegetable substance, and consequently exhibiting, on decomposition by heat, the phænomena characteristic of vegetable matter.—*Treatment:* If there be vomiting without severe pain or convulsions, copious ingestion of warm water may suffice: if no vomiting, it should be excited by mechanical irritation of the fauces and abundant administration of warm water or oil. These failing to induce sickness, *decoction of cinchona*, at the temperature of from 30° to 40°* should be immediately and largely given. The *yellow* bark has been found preferable to the red, as more powerfully decomposing the poison. Decoction of tea, of nut-gall with milk, of astringent barks and roots, may be employed when the cinchona is not at hand. The earths, alkalis, and sulphuretted hydrogen, are ineffectual or injurious. Opium may be given to allay excessive vomiting, particularly in nervous subjects. Local or general bleeding is indicated in constriction of the pharynx, or inflammation of the œsophagus, lungs, or stomach.

* 86—104 Fahrenh. Rev.

*Article* 4. *Species* 4. *Coppery Poisons.* Here ten varietes are enumerated. The second is *verdigris.* To it we shall restrict ourselves; as well fitted to illustrate the general characters, operation and treatment, of this important species of poisons.—It may, however, not be amiss to premise, that *metallic copper,* largely given, even in a state of extreme division, produces no injurious effects on the animal œconomy; that neither water, milk, tea, coffee, nor beer, on being heated, or allowed to remain, in a copper vessel, acquires deleterious properties, or dissolves an atom of the metal, provided it be free from verdigris;—that the metal is, on the other hand, powerfully acted on by water holding muriate of soda in solution; and that, lastly, if the solution of muriate of soda be previously mixed with beef, bacon, and fish, no such action on the copper vessel takes place. This extraordinary fact M. Orfila explains by supposing that these and "several other kinds of aliments destroy the effect of the solution of muriate of soda; and consequently cases of poisoning by food cooked in copper vessels, *not oxydated,* must be extremely rare."*

*Chemical history of verdigris.*† Its physical characters are well known. Pulverized and heated in a glass tube, it yields metallic copper and all the products furnished by vegetable substances when submitted to the action of caloric. It is decomposed by sulphuric acid with effervescence, and exhales the vapours of acetic acid. Boiling water dissolves the acetate of copper, but leaves a brown residuum containing the other constituent principles of verdigris. This solution, of a greenish-blue colour and strong styptic taste, reddens the preparations of litmus; is precipitated in the form of black sulphuret of copper, by sulphuretted hydrogen gas; sky-blue by potash and its subcarbonate; deep blue by ammonia (this precipitate dissolves on adding an excess of the alkali, and imparts to the solution a beautiful blue colour;) blue by barytes-water; green by liquid arsenious acid; fine yellow by chromate, and chesnut-brown by prussiate of potash. This last and ammonia are the most delicate tests. A cylinder

---

* The translator invariably renders the French verb, *doit,* and *doit être,* *ought* and *ought to be.* One short note, page 113, contains three examples of this false and inelegant construction. Rev.

† *Vert-de-gris,* Fr. *Impure sub-acetate of copper,* Br. According to Orfila, it consists of acetate, subacetate, and carbonate of copper, partly metallic, and partly oxydized, and vegetable and other foreign substances. Rev.

of phosphorus, and a clean plate of iron, on being immersed in the solution, decompose it, and acquire a coat of metallic copper. The liquid, in the former instance, loses its colour;* in the latter, becomes first green and finally red. With *tea*, the solution yields a reddish-yellow precipitate; with *albumen*, a blueish; with *milk*, a deep green coagulum; with *broth* and *gelatine*, no precipitate: in the latter instance, the mixture is affected by the ordinary tests, if employed in sufficient quantity, as the simple solution. Ammonia is useless as a test, if the poison be mixed with *red wine*; for then it produces a grey or black precipitate; but the hydro-sulphurets and prussiate of potash operate as on the plain solution. The blue precipitate, obtained by potash, yields, on calcination with charcoal, metallic copper: and the precipitate by albumen, and the coagulum from milk, give out to the action of caloric the metal mixed with the incinerated residue peculiar to animal substances. Verdigris, according to Drouard, *destroys life* by its direct operation upon the digestive canal, independently of absorption. Orfila, on the other hand, is induced to believe, from his own experiments, that death is the consequence of its introduction into the absorbent vessels and its action upon the nervous system. The lungs also, it is probable, participate the influence of the poison. Nine examples are cited, to illustrate the general train of *symptoms* marking its operation. These are acrid, styptic, and coppery taste; parched tongue, sense of strangulation; coppery risings; constant spitting, nausea, copious vomitings or fruitless efforts to vomit, rending pains of the stomach, violent colic, frequent stools, sometimes bloody and blackish, with tenesmus and debility; abdomen swollen and painful; pulse small, irregular, tight and frequent; syncope; heat natural,† intense thirst; dyspnœa; anxiety about the præcordia; cold sweats; urine scanty; violent head-ache, vertigo, depression,‡ weakness of the limbs, spasms, convulsions, death. All these symptoms, however, are rarely united: vomiting and colic are the most constant. Intestinal gangrene, when occurring, is announced by sudden cessation of pain, pulse nearly imperceptible, hiccough, and cold sweats. Inflammation and gangrene of the mucous, and sometimes other coats

* If there be a sufficient bulk of phosphorus.

† Incorrectly rendered, "*heat of the skin*," by the translator. Rev.

‡ Incorrectly, "*faintness*," in the translation. Rev.

of the stomach and bowels, with sloughs and perforations, are the *organic lesions* which characterize the action of verdigris.*

A reference to the chemical history of verdigris will be sufficient to direct *the conduct* of *the practitioner in different cases* of poisoning by this substance. We shall, therefore, merely observe, that the hydro-sulphurets, prussiate of potash, and ammonia; or affusion of sulphuric acid and calcination, are the tests principally to be relied on, according as the subject of examination is in a liquid or solid form; and that when no trace of the poison can be detected in the contents of the alimentary canal, metallic copper may frequently be obtained by submitting portions of the mucous membrane of the stomach and bowels to the action of strong heat in a crucible. This will particularly happen, when the membrane is blueish, hard, and firmly adherent to the muscular coat. *Treatment:* Hydrogenized sulphurets of potash, lime, and iron, extolled by Navier, as the antidotes to verdigris, are proved by Drouard to possess no such properties. They, like the saline and earthy alkalis, decompose it, but the product exerts an action equally deleterious. Infusion of nut-gall must also be rejected. "*Sugar and its preparations are the specifics against verdigris.*" This important fact is confirmed by numerous trials on man and other animals. Orfila proves, that this fortunate property is possessed by the sugar itself, and not by the liquid in which it may be dissolved: but what is the peculiar action exercised by sugar upon verdigris; or what the nature of the new compound, he has not yet been able to determine.† To a patient recently poisoned by verdigris, solid sugar and sugared water should, therefore, be given in large quantities: if these cannot be obtained, tepid or cold water, emollient decoctions or broth may be substituted. Vomiting, at the same time, should be provoked by irritating the fauces. These attempts failing, an emetic may be administered, particularly if there be oppression of the stomach without violent pains. Or, lastly, the stomach may be evacuated by the elastic gum tube. Vinegar should not be prescribed with this view. When the poison has been sometime swallowed,

* This sentence accords but ill with the preceding opinions of Orfila, on the physiological operation of verdigris. Rev.

† In an experiment with white sugar and verdigris, page 237, line 30, of the translation, ten *ounces* of the latter substance are erroneously put for ten *grains*. Rev.

copious vomiting taken place, and there are violent colics, vomiting must not be excited afresh: plentiful ingestion of cooling, oily, mucilaginous liquids, especially milk, by the mouth and rectum, is then to be employed and persisted in until the symptoms are relieved. Local and general bleeding, baths, and fomentations are the remedies indicated, if abdominal inflammation supervene: narcotics and antispasmodics will best allay the various commotions of the nervous system. An examination of the other eight varieties of coppery poisons concludes the *first part* of M. Orfila's first volume.

Part II. This part is occupied by an examination of the remaining species of corrosive poisons;* of the second or astringent class; and by an *appendix* upon iodine, and the antidotes of muriate of mercury, arsenic, and sulphuret of potash. Like the former, it is preceded by the report of Pinel and Vauquelin to the French Institute. This the English Translator has thought proper to omit.

Several poisonous substances are here considered, which can but rarely become the objects of medical observation or juridical inquiry. We shall, therefore, in general content ourselves with indicating their different species and varieties, and sketching rapidly their grand outline, as it regards their chemical composition and history; physiological action; internal and external phænomena displayed by them; practical application of this knowledge to the purposes of diagnosis and treatment. To all the more important species, we shall endeavour to give a proportionate developement.

*Article* 5. *Species* 5. *Tin.* Var. 1. *Muriates*; 2. *Oxydes.*—*Muriate of tin. Physical and chemical characters*: Colour yellowish white; taste styptic: composed of muriate at minimum, soluble; muriate at maximum, insoluble; and a ferruginous salt: partly volatilized by heat; partly soluble in distilled water. The solution, clear and colourless, reddens litmus; is not acted upon by *sugared water*; affords with muriate of mercury, *albumen, gelatine*, and prussiate of potash,† a white precipitate; sulphuric acid, yellowish white; muriate of gold, purple; hydro sulphurets, blackish; *tea*, and tincture of nut-gall,

---

* We obesrve *phosphorus* added to the list which was formerly given. Rev.

† The precipitate with prussiate of potash becomes blue on exposure to air. Rev.

clear yellow, *Burgundy wine*, violet: and it is powerfully decomposed by *milk* and *human bile*. *Physiological action*: Inflammation and corrosion of the organ with which the poison comes in contact. *Symptoms:* Rough metal ic taste; constriction of the throat; vomiting, severe abdominal pains, diarrhœa; slight dyspnœa; small, tight, frequent pulse; convulsions, sometimes paralysis, commonly terminating in death. *Organic lesions*: those characterizing the action of other corrosives, especially sublimate. *Practical application*: Analysis of the suspected liquids by the appropriate tests, and calcination of the solid poison; or of the alimentary mass, or mucous membrane with which it may be combined, so as to procure metallic tin. *Treatment*: *Milk* in very large quantities: if this cannot be obtained, broth; warm emollient decoctions. The usual remedies against abdominal inflammation, or nervous affections.

*Article* 6. *Species* 6. *Zinc. Var.* 1. *Sulphate;* 2. *Oxyde. Sulphate of Zinc. Phys. and chemical characters:* Colour white with yellow spots; texture grained; taste acrid and styptic; commonly mixed with sulphates of iron and copper.* Soluble in two parts and a half of water at 59 deg. Fah. The solution reddens litmus; is unaltered by *Burgundy wine* and *sugared water*; precipitated white by *albumen*; greenish white, by potash and ammonia; prussiate of potash, blue; chromate of potash, orange yellow; hydro-sulphurets, blackish; nut-gall and *tea*, deep violet blue: affords with *gelatine* and *human bile*, yellowish flakes; and curdles milk.—*Physiological action*: Mildest of all the metallic salts, from its tendency to excite vomiting: injected into the veins, it seems to induce death, by "stupifying the brain." *Symptoms:* those described in the last article, with the addition of paleness of face, and coldness of the extremities. *Organic lesions:* Slight inflammation of the intestinal mucous membrane; and sometimes extravasation of black blood on the muscular.—*Practical application, &c.* Suspected liquids are to be tried by the tests just enumerated; and efforts made to restore the poison to the metallic state. This difficult process may be effected by exposing the mass which contains it, previously dried and mixed with potash in a cru-

---

* This observation applies to the *impure* sulphate of zinc of commerce. When quite pure, it is precipitated white by ammonia, potash, and prussiate of potash; yellowish-white by the hydro-sulphurets: and tincture of nut-gall renders it milky without precipitation.

cible, for a long time, to a red heat.—*Treatment :* Vomiting to be excited by warm water, diluent drinks, milk, in large quantities: alkaline solutions are injurious: emollient injections, when the poison has passed the pylorus: abstraction of blood, if there be abdominal inflammation: opiates, if violent vomitings continue after expulsion of the poison.

*Article* 7. *Species* 7. *Silver.* Var. 1. *Nitrate of silver. Physical and chemical characters:* Colour white; taste bitter, acrid, caustic: decomposed when thrown on live coals, exhaling nitrous acid vapours, and yielding metallic silver: soluble in its own bulk of water at 59° Fah. The solution colourless, and imparting a violet stain to the skin, is not altered by ammonia, tincture of nut galls, or gelatine: precipitated white by muriatic acid, soluble muriates, and prussiate of potash; deep brown, by potash, soda, and lime-water; black, by hydro-sulphurets; carmine red (changing to purple on exposure to light) by chromic acid and chromate of potash; yellow, by phosphate of soda, arsenious acid, and the soluble arsenites, (the precipitate with the latter becoming black from the action of air): phosphorus and copper*, immersed in it, precipitate the metal:—on mixture with ten parts of *Burgundy wine,* it becomes violet, and is then precipitated white (changing, on the contact of air, into rose colour) by muriatic acid; greenish brown, by hydro-sulphurets; violet blue, by phosphate of soda:—with seven and a half parts of *tea,* it yields a deep purple red precipitate, inclining to black:—with fifteen parts of tea, is transparent, successively yellow, red, and black; and from this, muriatic acid throws down a yellow curdled sediment:—with *albumen,* it is precipitated white;—with nine parts of *broth,* yellowish white; with *bile,* orange yellow: lastly, it curdles *milk. Physiological action:* Introduced into the veins, it immediately destroys life, probably by its influence on the lungs and nervous system: swallowed, it seems to act by producing inflammation and corrosion of the texture in contact with it, and by the consequent epigastric re-action of the brain.—*Symptoms:* those of the other corrosives, sometimes with purple spots on the margins of the lips, and chin, and, perhaps, greyish white sloughs of the oral membrane.—*Organic lesions:* mucous

* In the former instance, phosphoric acid is the result: in the latter, nitrate of copper, and the liquid, of course, becomes blue.

membrane of the stomach converted into pulp or jelly,* or affected with redness varying in extent and degree; an appearance of scarification displayed in several points of its structure, and the colour of the sloughs greyish white, or very deep black, principally when the poison has been taken in a solid form; muscular coat highly inflamed and sloughy where the mucous is destroyed; sometimes complete perforations; mouth, pharynx, and œsophagus, sometimes showing similar morbid appearances.—*Practical application,* and *Treatment:* The liquids and solids to be analysed by re-agents and calcination in the usual mode. *Solution of muriate of soda in water is the antidote:* it must be promptly administered. Irritation should be soothed by diluent and mucilaginous liquids; and any consequent phlegmasia combated by the ordinary remedies.

*Article* 8. *Species.* 8. *Gold.* Var. 1. *Muriate;* 2. *Fulminating. Muriate of gold. Physical and chemical characters:* Chrystals of a deep yellow; taste styptic: thrown on live coals, it gives out muriatic acid gas and chlorine, and leaves metallic gold: soluble in water. The solution, of a variable yellow colour, stains the skin purple; reddens litmus; is unchanged by prussiate of potash; precipitated, by ammonia in small quantity reddish yellow; in excess canary yellow: deep chocolate, by hydro-sulphurets of potash, soda, and ammonia; reddish by nitrate of silver;—reddish yellow, by *tea*; chocolate, by tincture of nut-gall; fine purple, by *Burgundy wine* (in these two last examples pellicles of metallic gold float on the surface); yellowish, by albumen; in yellowish filaments by gelatine; precipitates milk in clots: human bile, added to a large quantity, throws down a green precipitate, changing to purple, and lastly to fine violet, if more be poured in.—*Physiological action:* less violent than sublimate, when taken into the stomach: corrosive, exciting inflammation: dreadfully destructive when thrown into the veins, and operating principally on the lungs. *Symptoms and organic lesions* in the human subject, little known. *Treatment:* that of the other corrosives.†

---

* The translator must needs have it "*boiled meat*" mistaking *bouillie* for *bouilli.* This negligence is unpardonable; since in many subsequent instances, he has rendered the same noun correctly. Rev.

† Our readers probably know that muriate of gold is employed on the Continent against syphilis. Dr Thompson proposes its decomposition by solution of sulphate of iron. Rev.

*Article* 9. *Species* 9. *Bismuth.* Var. 1. *Nitrate ;* 2. *Sub-nitrate.*—*Nitrate of bismuth. Chemical characters :* composed of acid nitrate, soluble in water, and sub-nitrate, insoluble. The former colourless, caustic, unpleasant, reddens litmus: largely mixed with water, it becomes turbid, throws down a little white sub-nitrate, and acquires greater acidity. Ammonia precipitates the solution white; the hydro sulphurets, black; prussiate of potash, yellowish white inclining to green ; tincture of nut-galls and *tea*, yellowish white; chromate of potash, orange yellow; *Burgundy wine*, (in the proportion of ten parts to one) violet; *albumen*, white ; the solution is undisturbed by *gelatine : human bile* separates it in yellow filamentous clots: it gives white coagula with *milk*.—*Physiological action* : introduced into the veins or stomach, it destroys life ; in the former case, by its influence on the nervous system; in the latter, by inflammation and corrosion of the stomach, affection of the lungs, and principally, where the result is rapid, sympathetic excitement of the brain. *Symptoms :* Pain; fearful anxiety ; vomiting, diarrhœa or constipation, colics ; heat in the chest ; fugitive shiverings ; vertigo; drowsiness*; sometimes dyspnœa, and convulsions before death. *Organic lesions* unknown in the human subject.—*Practical application &c.* The usual processes by chemical re-agents, and calcination with charcoal. Adulteration of wine by the oxyde may be detected by evaporating the fluid, and calcining the residue with charcoal to revive the metal ; of bread by exposing the mixture to a very elevated temperature in a crucible. —*Treatment : Milk ;* mucilaginous liquids : the ordinary resources, against abdominal inflammations.

*Article* 10. *Species* 10. *The concentrated acids.* Var. *Sulphuric ; nitric ; muriatic, &c. &c.* The observations of M. Orfila upon these poisons are so diffuse that we find it utterly impracticable to give an analysis of them equally rigorous as in the preceding articles : but though short, we shall endeavour to render it useful. While, however, on the one hand, wilful employment of these substances for the purpose of destruction is, at least among us, comparatively rare, and their sensible properties almost preclude the possibility of mistake ; so, on the other, from their peculiar operation on the living system, and strongly defined chemical characters, the juridical inquirer will in general be enabled to discriminate with facility their

---

* Incorrectly, "*faintings*" by the translator. Rev.

nature and combinations. With the *physical* properties of all these acids our readers must be sufficiently conversant. *Chemical*: *Sulphuric*, *nitric*, and *muriatic acids*, all agree in reddening litmus; destroying organized substances; producing little change on *gelatine*, *sugared water*, and *tea*; brightening or deepening the colour of *wine*; precipitating *albumen* white; *human bile* yellow;* and coagulating *milk* and *fluid blood*. *Sulphur. acid* precipitates acetate of lead white: its presence in *wine* and *vinegar* may be detected by the addition of chalk, and consequent formation of an insoluble sulphate of lime. *Nitr. acid*, treated with copper filings, exhales vapours of nitrous acid gas, and forms blue nitrate of copper: its presence in *vinegar* may be determined by saturating the fluid with potash, evaporating, and treating the residue with concentrated alcohol, which will dissolve the acetate of potash and leave the nitrate;—in *albumen*, by washing the precipitate, drying, and then boiling it in a solution of pure potash; the liquid will acquire a fine red colour, and furnish, on evaporation, a red-brown mass from which nitrate of potash may be separated by boiling in alcohol. *Muriat. acid* converts nitrate of silver into an insoluble muriate; does not render lime-water turbid; precipitates the soluble salts of lead, white: its existence in *wine* or *vinegar* may be ascertained by heating the mixture in a retort fitted with a receiver. The acid which will come over in the form of vapour and condense in the receiver, answers to the usual tests. This method is better than trial of the suspected fluid by the nitrate of silver. *Physiolog. action*: All these acids injected into the veins, prove suddenly destructive by coagulating the blood; taken into the stomach, by producing inflammation and disorganization of that viscus, and sympathetic commotion of the brain. *Symptoms*: Austere, acid, styptic taste; whole interior of the mouth often covered with white or black sloughs, which, after a time separating, excite irritative cough, and give the voice a croupy sound; burning heat from the fauces to the stomach; deep and acute pain in the throat; intolerably fœtid breath; excessive vomiting of a fluid sometimes black, sometimes red with blood, affecting the mouth with a bitter and styptic taste, and effervescing on a pavement; constipation or bloody stools; horrible pains

* Additional quantities of *sulphuric acid* renders the precipitate orange-yellow with light flakes of deep green;—of the *nitric acid*, successively green and brick-red;—of the *muriatic acid*, green.

in the abdomen and utter intolerance of pressure; pains in the breast, disordered respiration, anxiety; pulse frequent, small, concentrated, irregular, tremulous; skin constantly cold, horripilations; sometimes a papular eruption; physiognomy at first natural, afterwards completely changed; intellect undisturbed; dejection; restlessness, agitation, inability to remain in one posture, convulsive startings of the facial muscles. To this fearful groupe, several other symptoms are added from the testimony of Tartra, as characterizing ingestion of *nitric acid.* Such are, dysphagia, tenesmus, strangury; oral membrane of a dead white, thickened, and as though burned; surface of the tongue white or orange-coloured; teeth sometimes loose, their *coronæ* yellow. And, lastly, M. Orfila observes, that they who have swallowed *muriatic acid*, "exhale, in the first moments of the accident, a thick vapour, of a white colour, and very penetrating smell." *Organic lesions* from *sulphuric* and *muriatic acids:* Redness of the mouth, pharynx, and stomach; extravasations in the latter organ of venous blood; sloughs, perforations, sometimes ulceration, gangrene, or reduction into a kind of black pulp. *Nitric acid* is perhaps the only mineral poison, inflicting lesions of a peculiar character, and sometimes adequate, of themselves, to prove the nature of the case on simple anatomical inspection. These characters are, the yellow colour communicated to the lips, chin, and great part of the digestive canal; conversion of the mucous membrane into an adipose substance; perforations of the stomach, and effusion of a muddy yellow fluid into the abdomen.—After the chemical sketch of these substances, given in the present article, we think it needless to dwell upon the *application* of our resources to the various cases of poisoning by them. Carbonate of lime will be the best test of *sulphuric acid* in combination with alimentary substances; copper filings, or caustic potash, of *nitric acid;* and *muriatic acid,* if present in solid matter which has been vomited, may be recognized by boiling this matter with pure potash in distilled water, for three-fourths of an hour, and trying the fluid with nitrate of silver. The *Treatment,* in all these cases nearly the same, consists in throwing in large quantities of fluid containing *calcined magnesia, medicinal soap;* or, if these cannot be procured, milk, mucilaginous drinks or water may be substituted. The utmost promptitude and energy are required to save the life of the patient. *Oil* is sometimes useful in the first instance, as it excites vomiting. Abstraction of blood, and all the usual remedies, are indicated to obviate or subdue inflammation. The affection of the mouth should be treated as a local malady.

"If the nitric acid have been taken in small quantity, and there be reason to think that it has wholly entered into combination with the membrane of the first passages, the neutralizing plan should be abandoned, and diluting and emollient liquids very largely administered."

The chemical properties of seven other acids are also slightly traced here. These are the *phosphoric, nitrous, fluoric, sulphurous, phosphorous, oxalic*, and *tartaric*. But as the sketch contains nothing applicable to physiology, pathology, or practice, we shall not attempt its analysis.

We had hoped to include, in the present Number, the whole of M. Orfila's first volume. Our plan and limits, however, conspire to forbid it. In this analysis, it has been, and will be, our object to form a consistent and unbroken sketch, completely free from the obscurities of a foreign phraseolgy, unblemished by the sins of negligent translation; so concise as not to weary the most impatient of restraint, yet not too slight to possess value: such indeed as the busy practitioner, whose precarious hour of retirement admits not of deeper research, may consult with advantage, and such as will communicate to the intelligent pupil views and lessons with which his mind cannot be too soon or too strongly impressed. We are, therefore, unwilling to mutilate by ill-timed precipitation, what may prove an useful series of papers upon an important subject.

The pupil too, we think, may do well to go over, for himself, all those experiments with the poisons, which require only inexpensive materials and a simple apparatus. Thus his mind will become familiarized with the interesting phænomena of chemical affinity and decomposition; his hand acquire the dexterity which practice alone can give; and both be gradually trained to execute and comprehend the more delicate and complicated processes. And we can assure him, that with studies and researches like these, his vacant hour will be far more honourably and advantageously occupied, than with carrying on low intrigues in the kitchen; consulting his tailor upon the fashions; or sowing the seeds of lasting poverty and remorse by the indulgence of some puerile and romantic passion.

Chemistry is no longer the science of a deluded and visionary sect. Boundless in its empire, as in application to all the arts and purposes of civilized life, it has connections peculiarly striking and important with practical medicine. No system of general instruction can be comprehensive,—there can be no philosophic medical education, without it. Ignorance of it, in the gentleman, signally circumscribes the sphere of his knowledge, and intellectual enjoyments: in the practitioner of medicine, such ignorance is dangerous as disgraceful.

( To be continued. )

